# Multiplexed Prostate Cancer Companion Diagnostic Devices

**DOI:** 10.3390/s21155023

**Published:** 2021-07-24

**Authors:** Josephine Aidoo-Brown, Despina Moschou, Pedro Estrela

**Affiliations:** Centre for Biosensors, Bioelectronics and Biodevices (C3Bio), Department of Electronic & Electrical Engineering, University of Bath, Claverton Down, Bath BA2 7AY, UK; jga24@bath.ac.uk (J.A.-B.); d.moschou@bath.ac.uk (D.M.)

**Keywords:** prostate cancer, multiplex point-of-care testing (xPOCT), protein biomarkers, companion diagnostic devices

## Abstract

Prostate cancer (PCa) remains one of the most prominent forms of cancer for men. Since the early 1990s, Prostate-Specific Antigen (PSA) has been a commonly recognized PCa-associated protein biomarker. However, PSA testing has been shown to lack in specificity and sensitivity when needed to diagnose, monitor and/or treat PCa patients successfully. One enhancement could include the simultaneous detection of multiple PCa-associated protein biomarkers alongside PSA, also known as multiplexing. If conventional methods such as the enzyme-linked immunosorbent assay (ELISA) are used, multiplexed detection of such protein biomarkers can result in an increase in the required sample volume, in the complexity of the analytical procedures, and in adding to the cost. Using companion diagnostic devices such as biosensors, which can be portable and cost-effective with multiplexing capacities, may address these limitations. This review explores recent research for multiplexed PCa protein biomarker detection using optical and electrochemical biosensor platforms. Some of the novel and potential serum-based PCa protein biomarkers will be discussed in this review. In addition, this review discusses the importance of converting research protocols into multiplex point-of-care testing (xPOCT) devices to be used in near-patient settings, providing a more personalized approach to PCa patients’ diagnostic, surveillance and treatment management.

## 1. Introduction

Prostate cancer (PCa) is one of the most prevalent cancer types for men worldwide [1]. So far, prostate-specific antigen (PSA) has been considered to be an important biomarker for PCa diagnostic testing. In 1994, the use of a PSA screening test in combination with a digital rectal examination (DRE) was approved by the U.S. Food and Drug Administration (FDA) [2]. The PSA screening test is a standard clinical diagnostic test, comprised of a blood analysis for the quantification of PSA. According to established guidelines, serum PSA levels above 4 ng/mL provide an indication that PCa is present in an individual. However, PSA levels, particularly those between 4 and 10 ng/mL, are referred to as the diagnostic gray zone, in which elevated serum levels can be associated with other benign conditions, which can often be age-specific, such as benign prostatic hyperplasia (BPH) or prostatitis [3]. In addition to this, “normal” or “healthy” serum PSA levels (<4 ng/mL) can also be found in PCa patients. Therefore, the PSA test is lacking in both sensitivity and specificity for early detection of PCa. Leading to tentative misdiagnosis or needless and invasive prostate biopsies or radical prostatectomies for numerous PCa patients [4,5]. Other PSA derivatives have been calculated to improve PSA specificity, such as age-specific PSA cut-offs, percentages between free PSA and total PSA (%fPSA), PSA density (PSAD) and PSA velocity (PSAV) [5,6,7,8,9,10]. However, these attempts have not greatly increased the sensitivity and the specificity of the PCa diagnosis and treatment management. On the other hand, DRE testing normally has good specificity, e.g., DRE has been able to determine approximately 25% of clinically significant PCa patients who had been originally reported to display “normal” PSA serum levels [11,12]. However, this examination has a major drawback of variability depending on the experience of the examiner [12,13,14].

Because of the variations in the sensitivity and specificity of PSA and/or DRE tests, one significant research approach has been to simultaneously detect a panel of PCa protein biomarkers, also known as multiplexing [15,16,17,18,19,20,21]. Biomarkers for PCa can be identified in a variety of bodily samples, including prostatic tissue, serum and urine. Other biomarkers that could be examined include DNA methylation, microRNAs, circulating tumor DNA (ctDNA), metabolomics, volatile organic compounds (VOCs), and circulating tumor cells (CTCs) [10,22,23,24,25]. However, because of the widespread clinical and commercial ease of quantifying serum protein levels, especially when using traditional biomarker detection methods, this review concentrates on serum PCa protein biomarkers [10]. Potential PCa-associated protein biomarkers that have been identified (including those that have not been validated to date) for diagnostic, prognosis, and predictive stages, will be discussed in this review.

Conventional biomarker detection methods are used in clinical laboratory environments such as surface plasmon resonance, fluorescence analysis and enzyme-linked immunosorbent assay (ELISA) [26]. Of which ELISA is a typical gold-standard technique used for single-analyte detection of protein biomarkers retrieved from patient’s samples such as serum or urine [27]. However, conventional ELISA analysis is a lengthy and laborious process, requiring highly qualified professionals [28]. Furthermore, it is not a suitable method for a more reliable and tailored approach to PCa care and treatment management when trying to achieve precise, accurate outcomes while using a minute sample volume to detect several protein biomarkers [16,26,29].

Several biosensor platforms integrated with microfluidic systems have demonstrated multiple benefits when compared to ELISA, so that results can be obtained easily, requiring less steps and reducing costs [30]. This review aims to give an overview of the recent advances in biosensor systems that simultaneously detect multiple PCa-related proteins using optical or electrochemical detection techniques; it offers an insight into possible and effective integrated systems that can be translated into multiplex point-of-care testing (xPOCT) devices to be used in near-patient environments, such as hospitals, GP clinics or within patient’s homes.

## 2. Potential PCa Protein Biomarkers

Although PSA (human kallikrein 3, hK3) is a serine protease produced by epithelial cells inside the prostate gland, it is not specific to PCa [10,19,31,32]. Elevated levels of serum PSA may be caused by other factors than adenocarcinoma of the prostate. In addition, PCa can be present in men with low serum PSA levels (<4 ng/mL). However, as mentioned previously, PSA derivatives have been found to be of some clinical use to increase the sensitivity and specificity of PCa diagnosis [32,33].

Generally, multiple molecular isoforms of PSA circulate in serum [32,34]. For instance, approximately 70% of PSA can be found in serum as complexed PSA (cPSA), in which PSA is bound to serine protease inhibitors, such as α1-antichymotrypsin (ACT) and α2-macroglobulin [35,36]. About 30% of PSA do not form complexes with serine protease inhibitors, and are known as free PSA (fPSA), which in itself has several variants such as proenzyme PSA (proPSA) and intact or inactive PSA (iPSA) [36,37]. fPSA has been shown to be under-expressed in PCa patients relative to healthy patients or patients found to have benign diseases. Low fPSA serum levels have also been correlated with aggressive PCa [38]. Combinations of both cPSA and fPSA, with other PSA molecular isoforms is known as total PSA (tPSA) or simply PSA. tPSA and fPSA have been analyzed in combination, to calculate the %fPSA of patients with tPSA serum levels within the diagnostic gray zone (between 4 and 10 ng/mL). It has been shown that a higher %fPSA suggests lower probability of PCa on biopsy and raises the likelihood of an increase in PSA caused by BHP. However, both the %fPSA and PSA screening tests are constrained in their analytical performance to provide consistent diagnosis of PCa due to inter-assay variability [32,39]. In addition, more research into detecting multiple PSA isoforms using serum-based immunoassays has led to the development and commercialization of the Prostate Health Index (PHI; Beckman Coulter) and the 4-kallikrein score test (4Kscore^®^ Test; OPKO Health), both of which are used in clinical laboratories to help with the decision of whether an initial or repeat biopsy should be performed [5].

The Prostate Health Index (PHI) was approved by the FDA in 2012 for PCa diagnosis and active surveillance of PCa patients. The PHI compares serum protein levels of fPSA, tPSA, and a variant of pPSA called [−2]proPSA (or p2PSA) using the equation (p2PSA/fPSA) × tPSA^1/2^) [5,10,37,40]. The PHI test is intended for males over the age of 50 who have PSA values of less than 10 ng/mL, a normal DRE examination and are planning or reconsidering a prostate biopsy [41]. The PHI has been proven to have a diagnosis accuracy of 71% and a specificity of 26%, avoiding up to 40% of needless biopsies. Despite the fact that it has been demonstrated to outperform just evaluating fPSA or tPSA serum levels [5,10,37], the question of setting the cut off threshold for clinically significant PCa patients remains unanswered [40]. Furthermore, the 4-kallikrein score test (4Kscore^®^ Test) is a prediction model that uses laboratory analysis along with clinical characteristics such as age, prior prostate biopsy, and DRE results to provide the best prognosis for PCa patients [5,10,37,40]. The laboratory test comprises the measurement of four kallikrein proteins, including three PSA isoforms: fPSA, tPSA, and iPSA, as well as human kallikrein 2 (hK2), which is 80 percent homologous to PSA [10,37]. Overall, this test is for men who have high PSA levels and a positive DRE examination result. It has been found in multiple validation studies to eliminate needless biopsies while also being able to identify men with clinically severe PCa. However, there are certain restrictions on when this test can be performed. For example, it can only be conducted if men have not had a DRE in the last 96 h, or if they have not had any therapy or procedure for BHP symptoms [42]. Despite the fact that these tests have been thoroughly confirmed, research suggests that one method to improve their specificity is to select meaningful protein biomarkers other than PSA and related isoforms [10].

**Figure 1:**
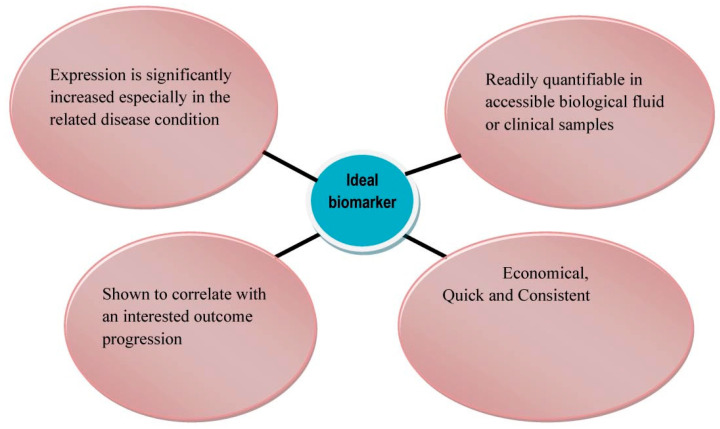
Desirable characteristics of ideal PCa-associated biomarkers. Reproduced with permission from ref. [43]. Copyright 2010 Ivyspring InternationalPublisher.

Currently, many emerging and potential PCa protein biomarkers have been identified (see Table 1), in particular relating to the diagnostic, prognostic or predictive stages of a PCa patient [16]. As illustrated in Figure 1, the function of a biomarker is to act as a biological indicator to determine any biological change that is contrary to normal biological conditions [10,43,44]. The main feature that needs to be taken into consideration are whether the biomarker has the ability to differentiate PCa from other benign prostatic conditions (diagnostic biomarker). In addition, PCa biomarkers should be able to provide a forecast discriminating insignificant or indolent PCa from clinically significant or aggressive PCa (prognostic biomarker). Furthermore, PCa protein biomarkers should be able to give insight of the likely patient response through active surveillance or during treatment (e.g., hormone therapy or chemotherapy), in order to proceed with the ideal treatment pathway (predictive biomarker) [10]. Unfortunately, it is very unlikely that a single biomarker will show all of these desirable characteristics, particularly because the majority of the biomarkers referred to in Table 1 of this review and in other respective reviews are not PCa-specific since they are associated with several other cancers or diseases. At the same time, however, simultaneously detecting multiple biomarkers instead of using single-analyte quantification methods may provide more accurate analysis within the diagnostic, prognostic and predictive stages of PCa [30,43,45]. Further developments are required to accurately determine the significance of each protein biomarker to be called a PCa-associated biomarker in order to efficiently predict PCa-related results, tailored for each PCa patient [43].

## 3. Multiplexed Detection of PCa Protein Biomarkers via Miniaturized Biosensor Systems

As previously stated, there has been a lot of focus on developing biosensors that detect numerous PCa protein biomarkers simultaneously, a process known as multiplexing, in order to improve decision-making during the critical PCa milestones (diagnostic, prognostic, and predictive stages). Antibodies are the most extensively used bioreceptor for detecting these protein biomarkers. When antibodies bind to their target antigen, whether via a sandwich or competitive assay, the overall set-up is referred to as an immunoassay [84]. Immunosensors involve the use of a biosensor platform in order to monitor the immunoassay from which binding events between antibodies and their respective PCa biomarker can be evaluated using several transducers such as mass-sensitive, electrochemical or optical transducers [84]. The focus of this section is to highlight current research achievements, demonstrating the sensitive simultaneous detection of multiple serum PCa protein biomarkers using optical and electrochemical transducers. Several optical detection protocols have been devised to quantify certain target analytes, including luminescence [85], surface-enhanced Raman scattering [86], and surface plasma resonance [87] detection techniques. Additionally, research has been performed on the application of amperometric, voltammetric and impedimetric methods for multiplex electrochemical biosensor detection. The advantages and disadvantages of using both optical and electrochemical techniques are further assessed in more detail by Roda et al. [88].

**Figure 2:**
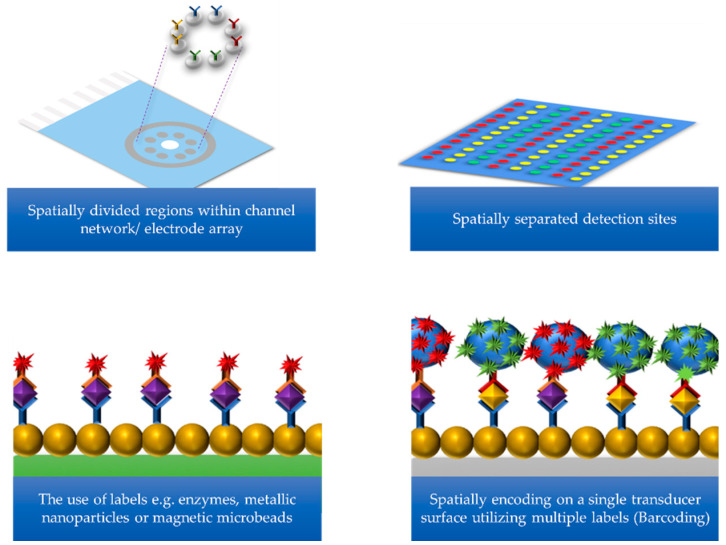
Schematic to multiplexing approaches in order to simultaneously detect multiple target analytes of interest.

Figure 2 describes various approaches to multiplexing, which include: the use of spatially separated detection sites for each biomarker; spatially divided regions within a channel network or electrode array; the use of several labels such as enzymes, metallic nanoparticles, or magnetic microbeads; and finally the use of spatially encoding on a single transducer surface using multiple labels (also known as barcoding) [16,20,89]. Some of these multiplexing approaches have been shown to improve the sensitivity of the biosensor during PCa protein detection and also have the potential to become commercially available xPOCT devices for PCa treatment management. For example, some of the research discussed includes the use of low-cost technologies to produce proof-of-concept prototypes or the integration of microfluidic systems to provide automated operation of biosensor platforms while requiring lower sample volumes [90,91].

### 3.1. Optical Detection Methods

#### 3.1.1. Luminescence

Luminescence generates a variety of cold light emissions, as it is not governed by the rising temperatures, as seen with incandescent detection platforms [92]. Thus, this type of optical detection technique generally involves an excited molecule that emits light energy while returning to its electronic ground state [93]. In terms of multiplexed detection of PCa protein biomarkers, the fluorescent, chemiluminescent and electro-chemiluminescent methods will be discussed below.

Fluorescence

Fluorescence is a type of photoluminescence that is initiated by the absorption of light energy (photons). This physical phenomenon is also known as photoexcitation [93]. Photodetectors are used to measure the changes in intensity after binding events of the target analyte and bioreceptor [94]. The labels only emit light at certain wavelengths when the analyte is found [30].

**Figure 3:**
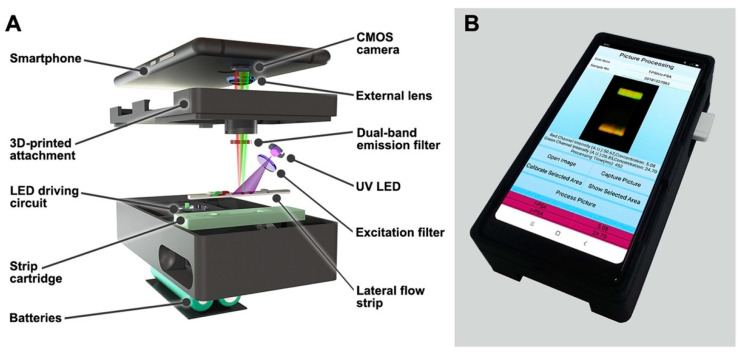
Smartphone-based dual-color fluorescent LFIA reader; (**A**) Internal structure of the smartphone readout device. MQB625 and MQB525 conjugates captured on the test line were excited by a 365 nm UV LED light source. Red and green emission signals passed through a dual-band emission filter (524/628 nm) and an external plano-convex lens, before reaching the smartphone CMOS sensor, (**B**) Depiction of the smartphone readout device. Reproduced with permission from ref. [95]. Copyright 2019 Elsevier.

Rong et al. developed a fluorescent lateral flow immunoassay (LFIA) using dual-color magnetic-quantum dot nanobeads (MQBs) to detect fPSA and cPSA simultaneously, as shown in Figure 3 [95]. Initially, protein biomarkers were attached to the capture antibodies modified with red (MQB625) and green (MQB525) colored MQBs, for fPSA and cPSA respectively, using an off-line capture protocol. After magnetic separation from unbound MQBs, the respective capture antibodies (anti-fPSA and anti-cPSA), were used as fluorescent detection probes. Subsequently, the sample solution was introduced to the LFIA, and using capillary forces, the sample solution migrated to the sensor surface immobilized with the monoclonal anti-tPSA detection antibodies to the nitrocellulose membrane, forming a sandwich format. Fluorescent images were analyzed using a dual-color strip readout integrated into a smartphone, as UV LED light stimulated the fluorescent detection probes attached to the protein biomarkers. The limits of detection for fPSA and cPSA in diluted fetal bovine serum were 0.009 and 0.087 ng/mL, respectively, within 1 h. Further to this, the LFIA was able to distinguish between clinical samples obtained from PCa and BPH patients when simultaneously detecting fPSA and cPSA in order to evaluate the %fPSA. Moreover, the clinical sample results were well correlated with the reference method, chemiluminescent microparticle immunoassay. It was concluded that the LFIA prototype could be specifically used as a xPOCT device in low-resource environments providing accurate diagnosis of PCa patients.

Chemiluminescence

Chemiluminescence (CL) is initiated by a chemical reaction between at least two luminescent reagents and is manipulated by the fluid flow [92,94,96]. The energy produced by the reaction of chemical reagents together causes the production of light [97]. The most common example of chemiluminescent detection involves the chemical interaction between luminol and horseradish peroxidase (HRP) [98].

For instance, Tang et al. have demonstrated the use of this detection technique to detect the PCa biomarkers PF-4 and PSA using an automated 3D-printed microfluidic array [99]. Using a touchscreen interface to operate the system’s pump, a sandwich format was constructed, by first immobilizing spotted arrays of poly L-lysine-coated glass slides with capture antibodies that bind to its respective protein biomarkers. This was followed by detection antibodies which were attached to several horseradish peroxidase labels (polyHRP) forming Ab_2_-polyHRP conjugates. The CL reagents were introduced to the detection chamber after the flow of the wash buffer. From which luminol reacted with hydrogen peroxide (H_2_O_2_) and was oxidized in the presence of HRP. The signal was measured using a coupled charged device (CCD) camera. A detection limit of 0.5 pg/mL was achieved for PF-4 and PSA in diluted calf serum within 30 min. The accuracy of the results was also confirmed by correlating results with ELISA assays using serum samples from non-PCa and PCa patients. Thus, it presents great opportunities to be used as a PCa xPOCT diagnostic device in resource-limited environments, because it is not only re-usable and fast but also cost-effective compared to the traditional ELISA.

**Figure 4:**
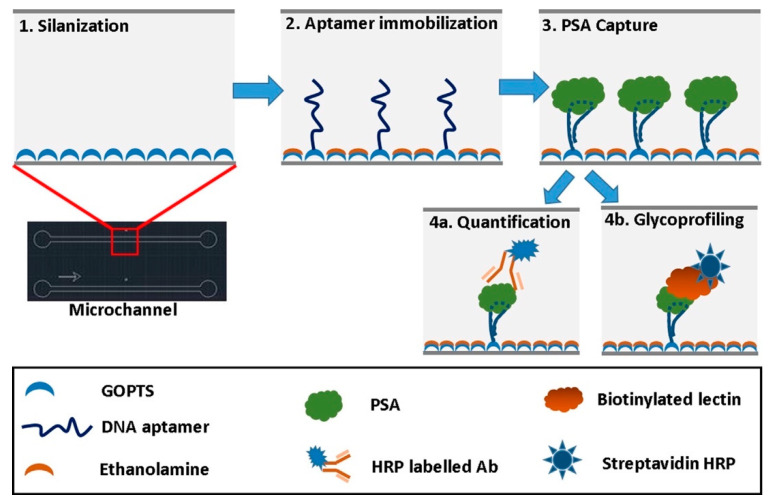
Illustration of microfluidic channel fabrication scheme for the quantification and glycoprofiling of fPSA. Reproduced with permission from ref. [100]. Copyright 2016 Elsevier.

Jolly et al. demonstrated an aptamer-based ELISA that replaced capture antibodies in a sandwich immunoassay with DNA aptamers for the quantification of fPSA and the glycoprofiling of fPSA [100]. It has been suggested that glycoprofiling of fPSA could be used to distinguish between indolent and aggressive forms of PCa. Thus, reducing unnecessary biopsies and further treatments that may have a negative impact on PCa patients [101]. A detection antibody, HRP-labeled anti-fPSA, was used for fPSA quantification within a single microchannel as shown in Figure 4. Whereas the parallel channel had the biotinylated *Sambucus nigra* (SNA) lectin (a biological protein) with a complementary streptavidin-HRP label, forming an aptamer-lectin assay for fPSA glycoprofiling. The optical changes that occurred during the binding of the respective receptors to fPSA were measured using a microfluidic CL sensor, via a microscopic CCD camera, after luminol had flowed into the respective microchannels. Detection limits for fPSA and fPSA glycans were 0.5 and 3 ng/mL in PBS, respectively. The detection limits are both relevant to the clinical ranges achieved using standard antibody-based immunoassays to evaluate the diagnosis or prognosis PCa patients [100].

Zhao et al. used a dual-labeled CL immunoassay to simultaneously measure tPSA and fPSA from diluted human serum samples in just over 1 h [102]. A sandwich immunoassay was also used, in which capture monoclonal antibodies were first immobilized on the sensing platform. However, two different labels, HRP and alkaline phosphatase (ALP), were used to differentiate between tPSA and fPSA detection monoclonal antibodies. The HRP-labeled antibody bound to both cPSA and fPSA was used to determine the amount of tPSA present in the sample. Whereas only fPSA was recognized by the ALP-labeled antibody. As a result, two chemiluminescence reactions occurred during the detection measurement as HRP reacted with luminol, ALP reacted with its respective CL substrate, 4-methoxy-4-(3-phosphate-phennyl)-spiro-(1,2-dioxetane-3,2′adamantane) (AMPPD). Detection limits of 0.03 and 0.05 ng/mL were found for tPSA and fPSA. The results obtained from this assay were also correlated with commercial chemiluminescent kits using clinical samples. It was concluded that this device would be useful for early diagnosis of PCa and could be used for routine clinical testing [102].

Electrochemiluminescence

In contrast to CL, electrochemiluminescence (ECL) is electrochemically generated, and therefore electron transfer at or near the working electrode is initiated and manipulated only after the application of the potential [94,96,98,103]. From which the light intensity emitted is detected due to the excited state of the reagents during the ECL reaction [92,96,104].

Sardesai et al. used the ECL to simultaneously detect PSA and IL-6 using a microwell single-wall carbon nanotube (SWCNT) immunoarray [105]. The SWCNT forests were situated within the hydrophobic polymer walls formed on a pyrolytic graphite (PG) chip inked with poly(butadine), in order to provide a conductive environment for ECL measurements. The array also consisted of a sandwich format with capture antibodies and detection antibodies. The detection antibodies were coated with tris(bipyridine)ruthenium(II) chloride ([Ru(bpy)_3_]^2+^) doped with silica nanoparticles (Ab_2_/RuBPY-SiNP). Both detection antibodies for PSA and IL-6 were bound to the same RuBPY-SiNPs in this study. To measure ECL, an electrolyte solution containing an ECL enhancer, tripropylamine (TrpA), initiated a chemical reaction with [Ru(bpy)_3_]^2+^ at 0.95 V vs. Ag/AgCl. Once the potential was applied, photoexcited [Ru(bpy)_3_]^2+^ was produced and was detected for 400 s using a CCD camera, only when an intensity of light was emitted at 610 nm. Detection limits were 1 pg/mL for PSA and 0.25 pg/mL for IL-6 in undiluted calf serum. Results using this array with patients’ serum also correlated with the ELISA single-protein analyte kits. Following this study, the same group adapted the microwell SWCNT immunoarray by integrating it with a microfluidic system for the detection of the same protein biomarkers (PSA and IL-6), which reduced the total assay time to just over an hour in comparison to three-hours when using non-microfluidic arrays [106]. The microfluidic system consisted of three molded polydimethylsiloxane (PDMS) channels which were situated on top of the chip and supported by a poly(methylmethacrylate) (PMMA) plate. The system also included a pump, a sample injector and a switching value for directing solutions to their respective channels. The authors achieved low detection limits for PSA (100 fg/mL) and IL-6 (10 fg/mL) in calf serum. The microfluidic device required only 2.5 µL of serum samples to preform triplicate analyses.

**Figure 5:**
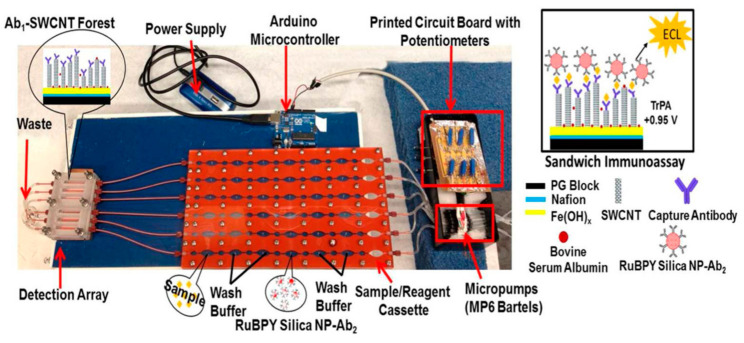
A microfluidic immunoarray with a 30-well detection array attached to PCB-controlled micropumps and sample/reagent cassette. The Arduino microcontroller is the microprocessor used to function the micropumps in order to perform the assay. Reproduced with permission from ref. [107]. Copyright 2015 American Chemical Society.

Kadimisetty et al. developed a 30-well microfluidic immunoarray using a low-cost automated microprocessor to detect four PCa protein biomarkers in less than 40 min [107]. The microprocessor was integrated with printed circuit board (PCB)-controlled micropumps, which were connected to six PDMS channels, as shown in Figure 5. SWCNT forests were also immobilized on the PG wafer to amplify the conductivity of the surface area. In this research protocol, RuBPY-SiNPs were coated with two antibody mixtures to form two duplex Ab2/RuBPY-SiNP detection labels, where label 1 was for PSA and IL-6 and label 2 for PSMA and PF-4. Within 36 min, low detection limits of 50, 100, 10 and 10 fg/mL were achieved for PSA, PSMA, PF-4 and IL-6 in undiluted calf serum, respectively. Excellent correlation was achieved with PCa patient serum compared to single-protein ELISA kits.

**Figure 6:**
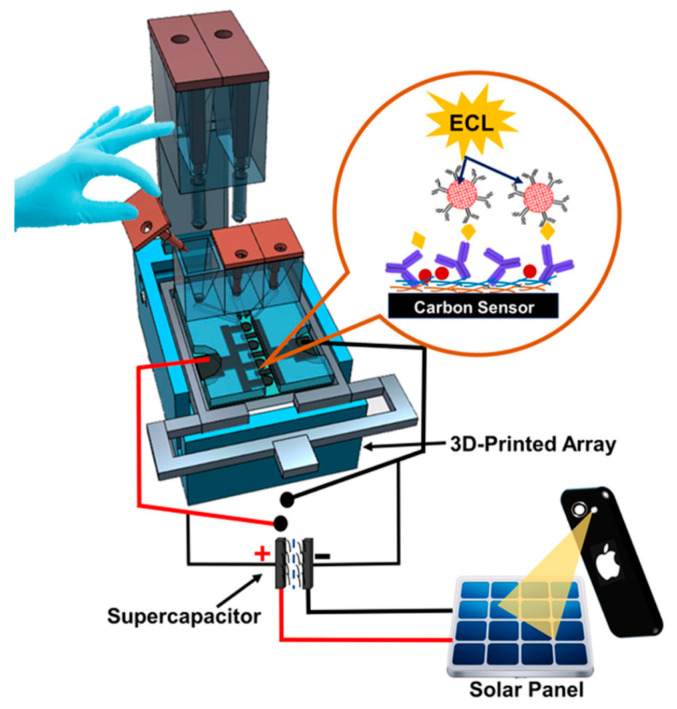
Illustration of 3D-printed supercapacitor-powered immunoarray using ECL detection technique. Reproduced with permission from ref. [108]. Copyright 2015 Elsevier.

Additionally, Kadimisetty et al. designed a 3D-printed supercapacitor-powered immunoarray to detect PSA, PSMA and PF-4 [108]. The supercapacitor was used to recharge the sensor system using solar cells between ECL measurements. The simplicity of the protocol reduced the cost of the immunoarrays’ materials, while achieving ultrasensitive detection within 35 min, which is comparable to their previous work. Detection limits of 300 fg/mL for PSA, 535 fg/mL for PSMA and 420 fg/mL for PF-4 were achieved in serum. The device, as depicted in Figure 6, could be used as a xPOCT in a low-resourced environment.

**Figure 7:**
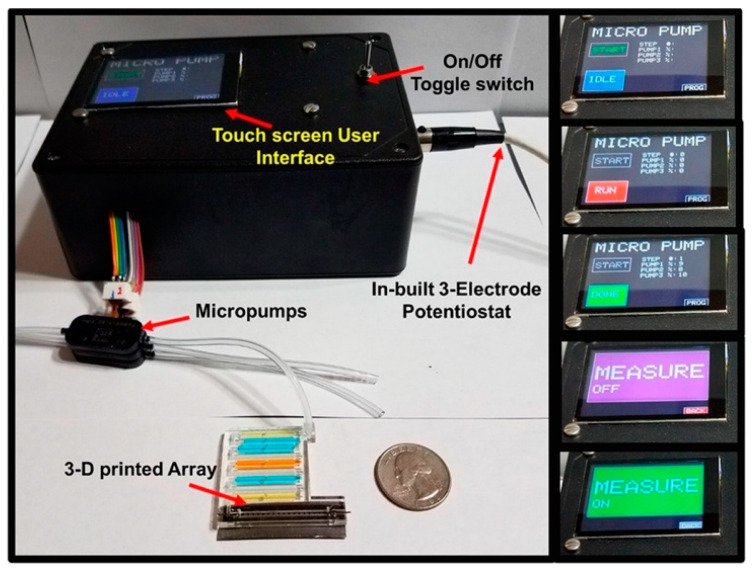
A 3D-printed immunoarray with touch screen user interface to control ECL measurements. A microfluidic array connected to a micropump is shown with dye-filled reagent chambers and graphite detection chip. Inset figures show multiple immunoassay steps along with messages to inform the user. Reproduced with permission from ref. [52]. Copyright 2018 American Chemical Society.

Further to this, Kadimisetty et al. developed another cost-effective 3D-printed immunoarray to detect eight potential PCa protein biomarkers via ECL. This included a 16 microwell detection chip, and a microfluidic system integrated with a user-friendly touch screen interface, as depicted in Figure 7. The touch screen interface was used to control the automated micropump which is connected to the microarray’s inlet port in to order to deliver samples and reagents in a timely manner [52]. The authors used the method mentioned earlier in the Sardesai et al. study [106], which involved the use of four duplex Ab_2_/RuBPY-SiNP detection labels (label 1 for PSA and PSMA, label 2 for VEGF-D and PF-4, label 3 for CD-14 and IGF-1, and label 4 for GOLM-1 and IGFBP-3). Once the detection antibodies have been attached to their target protein, TprA solution was introduced to the detection platform. The light intensity of the ECL reactions was measured using a CCD camera located in a dark box as the potential was applied. In undiluted calf serum, ultra-low detection limits between 110 and 500 fg/mL were achieved for PSA, CD-14, GOLM-1, IGFBP-3, IGF-1, PF-4, VEGF-D and PSMA within 25 min. The 3D-printed immunoarray exhibited accurate recovery percentages of approximately 100 ± 14%, while also achieving negligible antibody cross-reactivity between all eight proteins. The 3D-printed immunoarray could distinguish between non-PCa and PCa patients. Additionally, the authors suggested that this immunoarray could potentially be used to distinguish between clinically insignificant and significant PCa patients. However, more tests using human serum samples are needed to ensure that this is firmly concluded. Overall, it was deduced that the easy-to-use immunoarray is cost-effective, with xPOCT characteristics, especially when required in low-resourced environments [52].

#### 3.1.2. Surface-Enhanced Raman Scattering

Surface-enhanced Raman scattering (SERS) is another optical detection technique used to demonstrate the analysis of multiple protein markers because it is non-destructive, photostable and sensitive to the assessment of specific biomarkers [109]. SERS has been developed to increase the intensity of Raman scattering by a factor of up to 10^12^ [110]. The enhanced intensity is enough to measure sufficiently the changes in plasmonic resonance of molecules adsorbed singularly on or near roughened noble metal surfaces, such as gold (Au) or silver (Ag) [98,111,112,113,114]. Zhou et al. developed a multiplex SERS-based immunoassay that detected PSA, PSMA and hK2 [60]. A sandwich immunoassay was formed using silver nanoparticles (AgNPs) as the platform for the immobilization of the capture antibodies. The SiC/Ag/Ag-NPs SERS substrate was attached to the detecting antibodies to bind to the respective antigen. Using linear support vector machine (SVM) algorithms, the limits of detections for PSA, PSMA, and hK2 were 0.46 fg/mL, 1.05 fg/mL and 0.67 fg/mL, respectively. Additionally, they achieved 70% accuracy in distinguishing between PCa patients with BHP and healthy patients. The accuracy of the diagnosis of healthy or BHP patients was 75% and 60%, respectively. In comparison, 50% accuracy was achieved to detect only the serum level of PSA. This SERS platform could be used for diagnosing PCa patients in terms of providing clinical xPOCT.

Additionally, Chen et al. has developed a SERS-based vertical flow assay (VFA) for the detection of PSA, carcinoembryonic antigen (CEA) and α-1-fetoprotein (AFP) [115]. CEA and AFP are cancer protein biomarkers that are not specific to PCa as they can be found in a variety of cancer types [116]. Normally, conventional point-of-care VFAs are paper-based gold-conjugated immunoassays and gold colloids. Instead, Raman dyes (RDs) encoded core-shell SERS nanotags were used as detection probes to improve the precision and sensitivity during detection measurements. The authors have achieved detection limits of 0.37, 0.43, and 0.26 pg/mL for PSA, CEA and AFP respectively. It was concluded that this platform could be used as a xPOCT device, in conjunction with a portable Raman instrumentation, as a benchtop device in a hospital or GP clinic. This is because the SERS immunoassay provides highly sensitive biomarker detection meanwhile the VFA platform enables faster operation and simple analysis [115].

**Figure 8:**
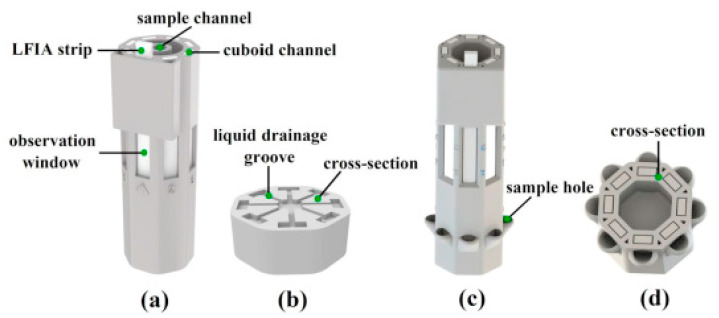
Detailed figure of the two types of multi-channel LFIA reaction columns; (**a**) Single-sample, multimarker reaction column (Type 1); (**b**) cross-sectional view of Type 1 LFIA column; (**c**) multisample, single-marker reaction column (Type 2); (**d**) Type 2 column’s cross-sectional view. Reproduced with permission from ref. [114]. Copyright 2020 Elsevier.

**Figure 9:**
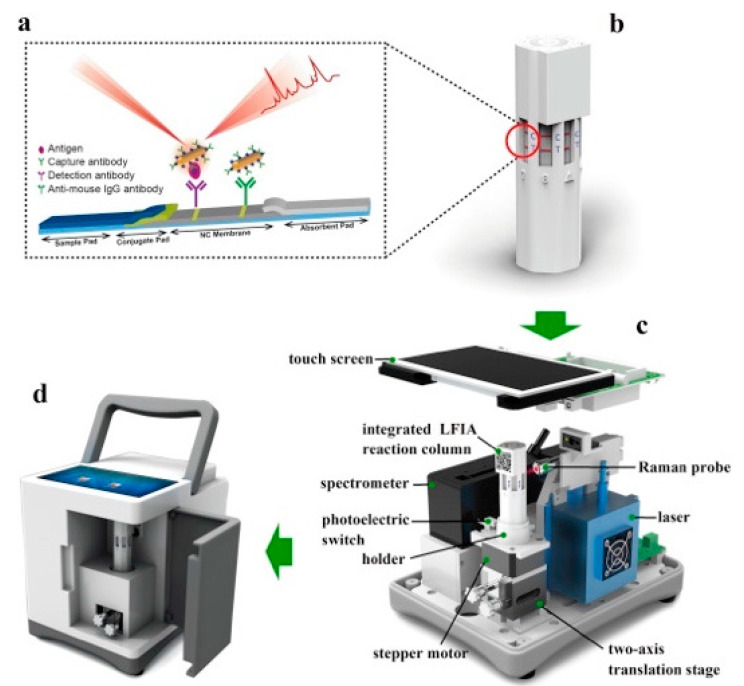
Illustration of portable SERS-based LFIA reader; (**a**) LFIA strip and (**b**) multi-channel LFIA reaction column, (**c**) Detailed schematic of the SERS-based LFIA reader and (**d**) the completed view of the portable reader. Reproduced with permission from ref. [114]. Copyright 2020 Elsevier.

In addition to this, Xiao et al. demonstrated the sensitivity and portability of a multiplex SERS-based immunoassay using an LFIA reader to also evaluate AFP, CEA and PSA [114]. The LFIA reader was 3D printed and could be incorporated between a choice of two multi-channel LFIA reaction columns, as shown in Figure 8. Type 1 of the LFIA column (single-sample, multimarker reaction column) involved the use of a liquid drainage grove which runs the same solution on all eight connected channels. Whereas the Type 2 LFIA column (multisample, single-marker reaction column) consisted of sample holders for each lateral strip, making it possible to detect a specific cancer biomarker on different strips within the same column. In Figure 9, either Type 1 or Type 2 of the LFIA column was placed on the holder inside the reader and operated via the stepper motor and two-axis translated stage to rotate back and forth and move up and down at specific times. Therefore, it was possible to perform SERS detection for each strip using the Raman probe to focus on the test and control lines, as targeted immunocomplexes were formed. In addition, the photoelectric switch corrected the position of each observation window to ensure that measurements were carried out in an orderly manner. The SERS nanotags used were composed of gold nanorods (AuNRs) and a Raman reporter molecule, 5,5′-dithiobis-(2-nitrobenzoic acid) (DTNB), to match the laser wavelength of the excitation (785 nm). These AuNR−DTNB plasmonic NPs were further functionalized with specific detection antibodies and BSA, which provided biocompatibility and stability. The Type 1 column was used for evaluating the LFIA reader’s specificity in which five antibodies were immobilized on the control line of the strip. This involved the use of all three cancer biomarkers’ antibodies as well as the antibodies specific to interfering inflammation biomarkers, C-reactive protein (CRP) and Procalcitonin (PCT). The test line was found to visually darken only when the target cancer biomarker was captured, regardless of the addition of interference protein biomarkers. The visual interpretation of the test lines also corresponded to the SERS signal detected. In addition, uniformity tests have shown that the device produces uniform, reliable and stable results. When using the Type 2 column, the overall process, which involved 20 repeats for each of the eight strips used, took place within 18 min. Detection limits of 0.01 ng/mL in PBS solution for all three cancer biomarkers were achieved, which was 1000 times more sensitive than that acquired for visual signals. Lastly, clinical serum samples consisting of either positive or negative samples were correctly detected for all three cancer biomarkers using the Type 2 LFIA columns. The results of the clinical sample were also well correlated with ECL and demonstrated higher sensitivity compared to the ELISA analysis. Overall, the SERS-based LFIA has the potential to be used as a xPOCT device in multiple applications including the diagnosis and treatment management of PCa patients.

An overview of recent developments of optical biosensors for multiplexed detection of PCa protein biomarkers is presented on Table 2.

### 3.2. Electrochemical Detection Methods

#### 3.2.1. Amperometric Techniques

Amperometry focuses on measuring the resulting current as a constant potential is applied within the electrochemical cell [117,118]. Amperometric detection was used by Chikkaveeraiah et al. to achieve multiplexed detection of PSA, PSMA, PF-4 and IL-6 [68]. Single-wall carbon nanotube forests (SWCNF) have modified the four working electrodes. Each working electrode was then immobilized with one of the four respective capture antibodies. After the capture antibodies bound to the protein biomarkers, they are attached to detection antibodies to form a sandwich immunocomplex. PSA and PSMA detection antibodies have been modified with HRP, whereas streptavidin-HRP (SA-HRP) labels were used to modify the PF-4 and IL-6 biotinylated detection antibodies. Approximately 16 SA-HRP labels were attached to a single antibody in order to achieve a higher sensitivity while detecting an electrochemical signal. This significantly amplified the amperometric signal detected during binding events for PF-4 and IL-6, as their protein dynamic concentration ranges are lower compared to PSA and PSMA. In the presence of the mediator, hydroquinone, HRP catalyzed H_2_O_2_ producing specific amperometric reduction peaks at a voltage of −0.3 V, while detecting different concentrations of the respective biomarker. This resulted in detection limits of 1, 10, 1 and 0.03 ng/mL for PSA, PSMA, PF-4 and IL-6 in diluted calf serum, respectively [68].

Moreover, using amperometric detection, Chikkaveeraiah et al. were able to achieve lower detection limits of 0.23 pg/mL and 0.30 pg/mL for PSA and IL-6, respectively, in diluted calf serum using a PDMS microfluidic-based platform [15]. Capture antibodies were immobilized on eight working electrodes that had previously been deposited with glutathione-decorated gold nanoparticles (GSH-AuNPs). In this study, superparamagnetic nanoparticles conjugated with specific detection antibodies (~90,000 per nanoparticle) and HRP labels (~20,000 labels per nanoparticle) were used for the off-line capture of the PCa protein biomarkers in calf serum solution. Using a syringe, the modified superparamagnetic nanoparticles solution flowed to the respective electrode surfaces. Following this, hydroquinone and H_2_O_2_ solutions were introduced to initiate an electrochemical reaction that could be detected amperometrically. Additionally, the biosensor platform was well correlated with ELISA in the use of patient serum samples, while also dramatically reducing manufacturing costs compared to conventional systems. A faster total analysis time (1.15 h) was achieved, and a minute sample volume (5 µL) was required to obtain highly sensitive and specific results.

Sharafeldin et al. used an offline capture method within a microfluidic electrochemical immunoassay to simultaneously detect PSA and PSMA in undiluted calf serum [119]. In this case, the working electrodes were modified with iron oxide nanoparticles (Fe_3_O_4_ NPs) on graphene oxide nanosheets, which were then decorated with specific capture antibodies using 1-(3-(dimethylamino)propyl)-3-ethylcarbodiimide hydrochloride (EDC)/N- hydroxysulfosuccinimide (NHS) chemistry. The microfluidic system was used to introduce serum samples to initiate the respective binding events of the immobilized capture antibodies to their specific protein biomarker. The bound protein biomarkers were magnetically separated from the unbound biomarkers in the sample. From which the solution flowed through the microfluidic system to the detection chamber, where the detection antibodies attached to their respective protein biomarker. Fe_3_O_4_ NPs performed similarly to HRP, capable of catalyzing hydrogen peroxide to produce an amperometric signal. Detection limits of 15 and 4.8 fg/mL were achieved for PSA and PSMA, respectively. The results were comparable to previous studies using detection antibodies modified with magnetic beads and HRP. Moreover, the immunoassay was well correlated with the ELISA method when using patient serum samples.

**Figure 10:**
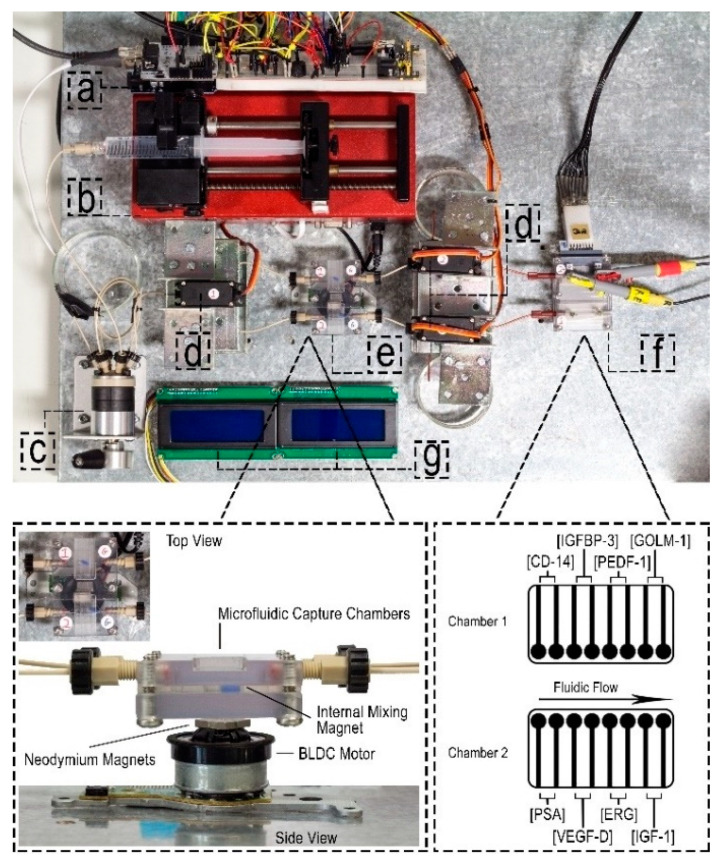
Illustration of an automated microfluidic immunoarray platform, featuring; (**a**) Arduino Uno microcontroller, (**b**) syringe pump, (**c**) sample injector, (**d**) servo-actuated valves, (**e**) capture chambers and magnetic stirrers, (**f**) detection chambers, and (**g**) LCD displays. Reproduced with permission from ref. [55]. Copyright 2019 Wiley-VCH Verlag.

Mercer et al. developed a microfluidic immunoarray platform, powered by a programmable Arduino microcontroller, capable of detecting eight PCa protein biomarkers simultaneously, negating the need for a desktop or laptop [55]. Using the protocol described by Otieno et al. [120], the carbon working electrodes were modified with a layer of poly(diallyldimethylammonium chloride) (PDDA), followed by GSH-AuNPs. The modified electrodes were then immobilized with sandwich immunocomplexes, consisting of MP-Ab2-HRP conjugates as the detection antibodies. The immunoarray exhibited two amperometry protein detection chambers, as depicted in Figure 10. The Arduino microcontroller powered the automated processes of the microfluidic system, incorporating several components, including valve actuators, a syringe pump, magnetic stirrers and an electronic display. Amperometric detection limits of 140, 90, 15, 13, 130, 150, 90 and 15 pg/mL were achieved in serum for PSA, VEGF-D, erythroblast transformation specific related gene (ERG), IGF-1, CD-14, IGFBP-3, pigment epithelium-derived factor (PEDF-1) and GOLM-1, respectively, within 30 min [55]. ERG is over-expressed in patients with PCa and contributes to PCa progression [2,121], whereas PEDF is suggested to exhibit down-regulated serum levels in PCa patients, acting as an angiogenesis inhibitor. Overall, the microfluidic immunoarray platform demonstrated the possibility of being used as a clinical xPOCT device, such as a hospital clinic, suitable for diagnosing and staging PCa progression [55].

#### 3.2.2. Voltammetric Techniques

**Figure 11:**
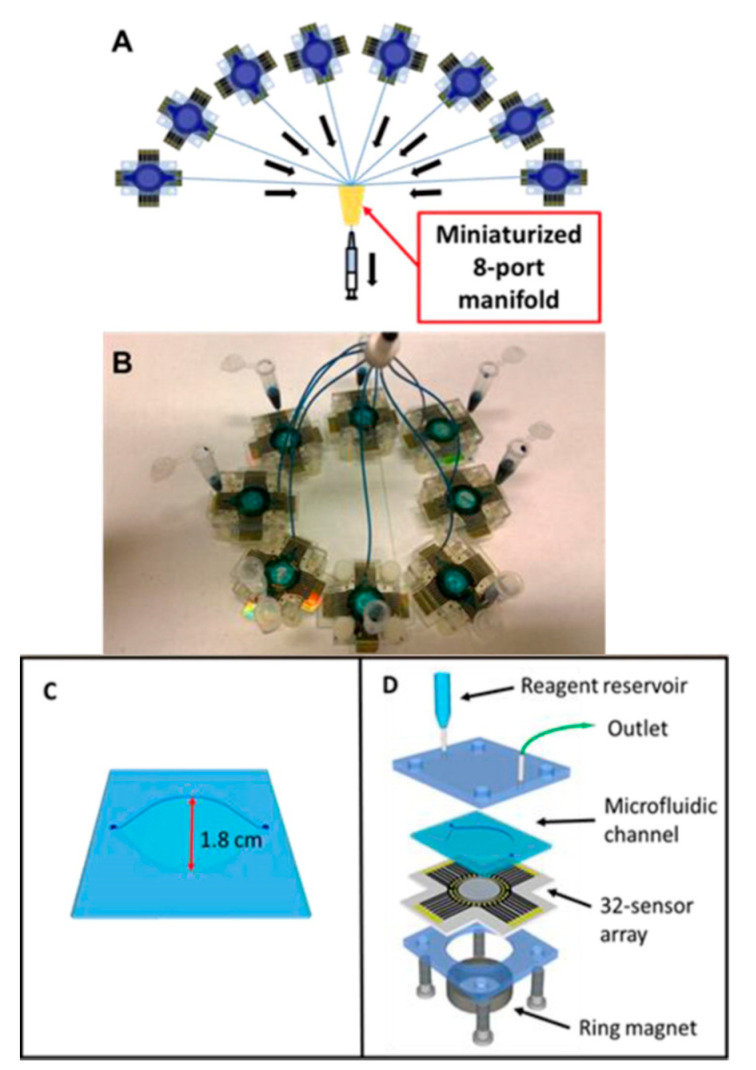
An electrochemical microfluidic immunoarray: (**A**) 256 individual working microelectrodes configuration; (**B**) 8 microfluidic immunoarrays are connected via miniaturized 8-port manifold; (**C**) molded PDMS microfluidic channel, and (**D**) deconstructed view of the integrated microfluidic immunoarray. Reproduced with permission from ref. [123]. Copyright 2016 American Chemical Society.

Voltammetry is a sub-class of amperometry which measures the flow of electrons as a varied potential is swept across the working electrode [118,122]. Tang et al. devised a cost-effective electrochemical microfluidic immunoarray containing eight miniaturized ports in order to achieve 256 individual working microelectrodes, simultaneously detecting PSA alongside PSMA, IL-6 and PF-4, within one hour [123]. It was noted that each immunoarray contained 32 sensors, which were divided into four sections, for the respective protein biomarkers, as depicted in Figure 11. For simplicity during the DPV measurements, each 32-sensor array had its own on-chip reference and counter electrodes. SAM modified electrodes were attached to the hydrophobic wells to prevent cross-contamination of the antibodies on their respective surface during immobilization. Furthermore, the SAM layer, composed of mercaptopropionic acid (MPA), was immobilized on the electrode surface, followed by the attachment of capture antibodies using EDC/NHS chemistry. An off-line capture protocol was established within a separate reservoir to attach different protein concentrations to their respective biotinylated detection antibodies that were functionalized in conjunction to biotinylated HRP labels (~8500 HRP labels per nanoparticle onto streptavidin coated magnetic nanoparticles). From which bound detection antibodies were introduced to the microelectrodes using the microfluidic system by means of an inlet tube connected to the reagent reservoir. To load the reagents into the microfluidic system, a syringe was connected to the outlet tubing of all eight of the immune arrays, effectively detecting the protein biomarkers. Hydroquinone and H_2_O_2_ were introduced to the microfluidic system for the measurement of differential pulse voltammetry (DPV). For six replicates of eight protein concentrations, the limits of detections for PSA, PSMA, PF-4 and IL-6 in diluted calf serum were 2, 0.15, 0.1 and 0.05 pg/mL, respectively. This immunoarray demonstrated high-throughput detection of multiple protein biomarkers at a low cost, using simple but highly sensitive equipment.

Pan et al. also used DPV to detect VEGF and PSA from serum samples of PCa patients simultaneously [124]. A three-step fabrication process involving metal-film deposition, photolithography and metal etching was used to develop the two-electrode system consisting of gold working and counter electrodes on a glass slide. For the completion of the three-electrode system, a separate silver/silver chloride (Ag/AgCl) reference electrode such as. The graphene oxide modified working electrode was immobilized with VEGF-specific DNA aptamers. The electrode surface was then introduced with the VEGF solution. Similarly, to the CL protocol of Jolly et al. [100], detection antibodies were then introduced to the surface of the sensor. However, PSA and VEGF were analyzed on the same sensor surface instead of using parallel microchannels. Detection antibodies for PSA and VEGF (anti-PSA and anti-VEGF) were functionalized onto modified poly-L-lactide nanoparticles (PLLA NPs) and then introduced to the electrode surface. In which the anti-VEGF antibodies on the PPLA NPs bound to the VEGF protein immobilized on the electrode surface to form a sandwich-based assay. After this, the biosensor was immersed in PSA solution that also bound to the anti-PSA antibodies present on the PLLA NPs. This resulted in detection limits of 50 pg/mL and 1 ng/mL for VEGF and PSA, respectively, and highly correlated with ELISA in the evaluation of samples from early staged PCa patients.

**Figure 12:**
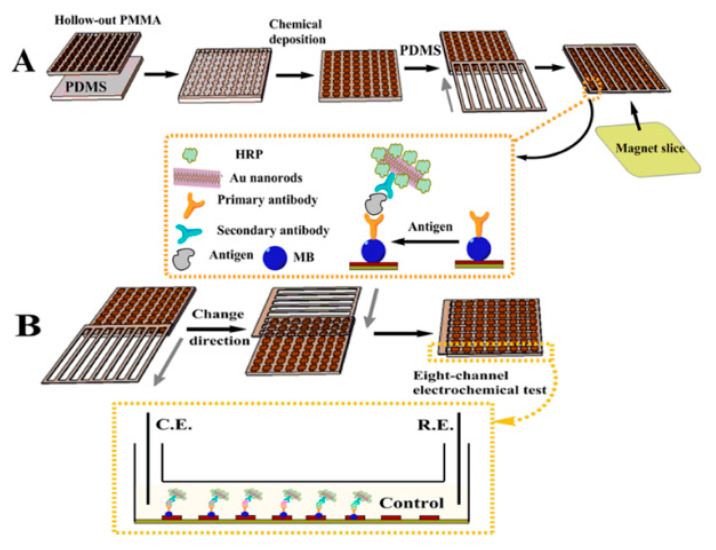
A flexible PDMS 8 × 8 electrode immunoarray; (**A**) Schematic of preparing the microchip along with the sensor surface modifications; (**B**) Illustration of the detection of PSA, PSMA and IL-6, with control measurements, in one microchannel. Reproduced with permission from ref. [69]. Copyright 2014 American Chemical Society.

Alternatively, square wave voltammetry (SWV) was used by Akbari Jonous et al. to simultaneously detect tPSA and fPSA, using a carbon working electrode with a sandwich-based format [125]. Reduced graphene oxide and AuNPs were used to modify both the capture and detection monoclonal antibodies. This significantly magnified the voltammetric detection signal as nanomaterials increased the carbon electrode’s conductivity, resulting in faster electron transfer rates. Detection limits of 0.2 and 0.07 ng/mL were determined for tPSA and fPSA, respectively. The biosensor was well correlated with the standard CL test using patients’ serum samples. The authors suggested that it could be used as a PCa diagnostic POCT device. Additionally, Liu et al. have developed a flexible PDMS 8 × 8 electrode immunoarray for multiplex electrochemical detection of PSA, PSMA and IL-6, as shown in Figure 12 [69]. The Au electrodes were used to form a sandwich-based immunosensor, initially immobilized with capture antibodies functionalized with magnetic beads. In addition, detection antibodies were functionalized with AuNRs decorated with HRP (HRP-Ab_2_-AuNRs). Cyclic voltammetry (CV) was used to measure the resulting current with low detection limits of 0.1, 0.8 and 0.005 ng/mL determined for PSA, PSMA and IL-6, respectively. The authors concluded that the microchip could be used as a xPOCT device, exhibiting strengths due to its versatility, in terms of fabrication, modification processes, and storage. It is also less likely to be damaged compared to rigid glass substrates used for the fabrication of biosensor platforms.

#### 3.2.3. Impedimetric Techniques

**Figure 13:**
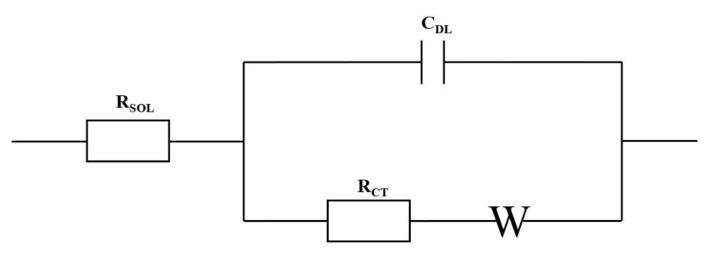
Randles equivalent circuit to the model the charge flow during electrochemical impedance spectroscopy detection, R_CT_ represents the charge transfer resistance, R_SOL_ denotes the uncompensated solution resistance, C_DL_ represents the capacitance and W signifies the Warburg diffusion coefficient.

Impedance-based biosensors are label-free and highly sensitive electrochemical detection techniques that reduce the number of reagents required and hence reduce the overall analysis time [4,94]. Electrical impedance spectroscopy evaluates the capacitive or resistive behavior established from the charges separated at the electrode-electrolyte interface [94,117]. Impedance-based biosensors require the application of a low sinusoidal ac voltage (typically 5–10 mV) at a specific frequency [94,118]. The voltage perturbation is used to demonstrate the biological binding events that occur at the surface of the electrode by evaluating the charge flow [117]. The charge flow or electrical signal can be modeled using the Randles equivalent circuit, as depicted in Figure 13 [126]. The charge resistance at the interfacial layer of the working electrode is known as the charge transfer resistance, R_CT_. Within the circuit, the R_CT_ is parallel to the capacitance, C_DL_, which describes the electrode-electrolyte interface’s electrical double layer. Additionally, in series to the R_CT_, W represents the Warburg diffusion coefficient and R_SOL_ is to demonstrate the uncompensated resistance of the solution [127]. Impedance data are generally represented using Nyquist or Bode plots [128].

**Figure 14:**
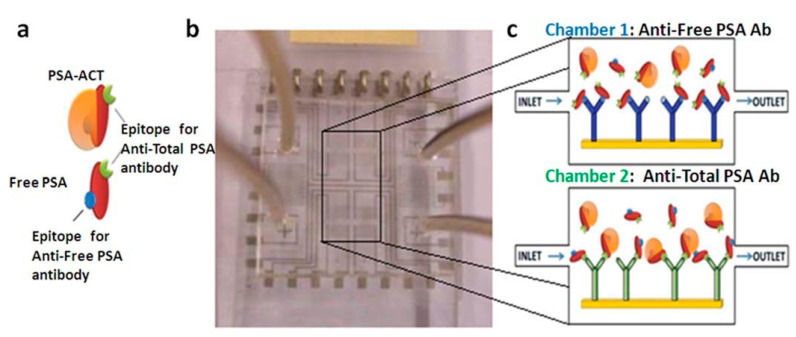
(**a**) Schematic representation of PSA antigens-related (cPSA and fPSA) and (**b**) device composed of two chambers for detecting the antigens: (**c**) one chamber is functionalized with anti-fPSA antibodies (Chamber 1) and the other one with anti-tPSA antibodies (Chamber 2). Reproduced with permission from ref. [129]. Copyright 2013 Royal Society of Chemistry.

Chiriacò et al. designed an impedimetric dual-labeled microfluidic biosensor based on the conventional ELISA method [129]. The PDMS-based microfluidic system had two chambers, as shown in Figure 14. Each chamber consisted of modified SAM electrodes with anti-fPSA and anti-tPSA antibodies, respectively, using EDC/NHS chemistry. Anti-tPSA capture antibodies could be attached to both biomarkers, fPSA and cPSA, both of which have the same epitope recognition on their surfaces. This provided accurate %fPSA measurements, therefore, in order to diagnose and also to distinguish PCa patients from other conditions. The fPSA and cPSA solutions flowed into their respective chambers. A solution containing the electrochemical redox probe, [Fe(CN)_6_]^3–/4–^, was then used during electrochemical impedance spectroscopy (EIS) measurements. It was found that following the functionalizing of the electrode with the SAM layer and subsequent binding of the antibodies to their respective biomarker, the R_CT_ value increased. The electron transfer from the bulk solution to the working electrodes was therefore restricted. Sensitive detection limits of approximately 1 ng/mL in PBS solution were achieved for both biomarkers. Additionally, the authors were able to simultaneously detect fPSA and cPSA in order to evaluate %fPSA. If the %fPSA is lower than cut-off level (<25%), this determined that the patient has PCa. Using two solutions with fixed %fPSA (50 and 20%), the biosensor was able to distinguish between the two solutions, calculating the %fPSA to be 42% and 19%, respectively. This platform therefore has the potential to distinguish BHP patients from PCa patients. Thus, using this simple fabrication process, the overall analysis time has not only been shortened compared to the conventional ELISA method, but is also cost-effective using a simple manufacturing process.

Additionally, Pihíková et al. reported a label-free impedimetric biosensor capable of detecting PSA and PSA glycans at the same time [130]. As mentioned previously, research has been conducted on how changes in the conformation of PSA glycosylation can be linked to PCa progression. Primarily, the capture antibodies were immobilized to the SAM layer, composed of 11-mercaptoundecanoic acid and 6-mercapto-1-hexanol, on the surface of the sensor, using EDC/NHS chemistry. This allowed the PSA to attach to the capture antibodies. Mass spectrometry is typically used for the analysis of PSA glycans. However, the lectin, SNA, was used by the impedimetric biosensor to detect glycosylated PSA forms. The lectins specifically attached to the terminal sialic acid present on PSA, to form a sandwich format. [Fe(CN)_6_]^3–/4–^ was used as an electroactive redox probe for EIS measurements. Sensitive detection limits of 4 aM (about 0.13 fg/mL) were achieved for both PSA and PSA glycans.

**Figure 15:**
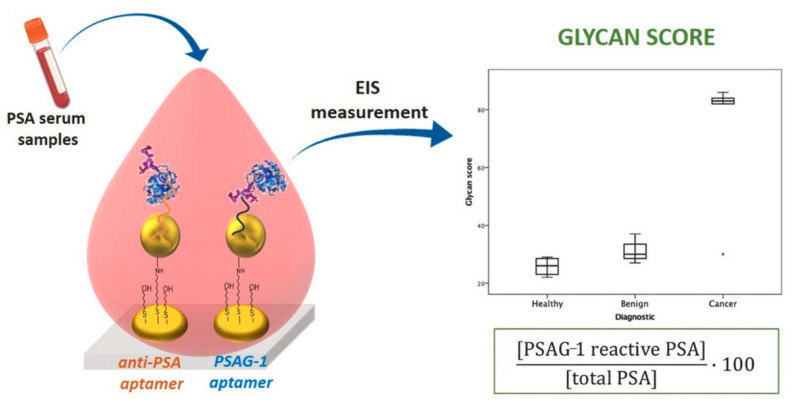
Illustration of anti-PSA and PSAG-1 aptamers used in the dual aptamer-based impedimetric biosensor to detect PSA and PSA glycans. Using clinical serum samples, the EIS measurement were used to measure the glycan score (GS), which is the ratio between the concentration of the glycosylated PSA (detected with PSAG-1 aptamer) to tPSA (detected with anti-PSA aptamer), multiped by 100. According to the graphical data, the GS can be used to distinguish known PCa patients from benign and healthy patients. Reproduced with permission from ref. [131]. Copyright 2020 Elsevier.

Diaz Fernandez et al. developed a dual aptamer-based impedimetric biosensor, comprised of two adjacent nanostructured gold electrodes that detected both PSA and glycosylated PSA (PSAG-1) using anti-PSA and PSAG-1 aptamers [131]. A SAM layer of 11-amino-1-undecanothiol was applied to the gold working electrodes. Mercaptohexanol (MCH) was used as a blocking agent or backfiller before AuNPs were introduced to immobilize the SAM layer. After that, another SAM layer was applied to the surface, consisting of a 1:100 ratio of the relevant aptamer (anti-PSA/PSAG-1) and MCH. In diluted serum, the aptasensor had detection limits of 0.64 and 0.26 ng/mL for PSA and PSAG-1, respectively. When impedimetric detection with the PSAG-1 aptamer was used to detect recombinant PSA (rPSA), the signal rise was lower than when detecting human PSA (hPSA). When using the anti-PSA aptamer to detect both rPSA and hPSA, a similar signal increase (96%) was seen between the two PSA proteins. As a result, PSAG-1 has been confirmed as the aptamer that can recognize PSA’s glycosylated sites. Human serum albumin was shown to have a very little interference, indicating that this platform is overall selective to PSA. Furthermore, the glycan score (GS) was calculated using clinical serum samples. The GS is the ratio between the concentration of the glycosylated PSA (detected with PSAG-1 aptamer) to tPSA (detected with anti-PSA aptamer), multiped by 100. In comparison to benign and healthy patients (values between 22 and 37), a clear correlation was discovered between the GS and the known diagnosis of PCa patients (values between 82 and 86), as illustrated in Figure 15, rather than looking at the concentrations of the analytes independently. The authors came to the conclusion that this platform might be used to improve patient PCa diagnosis while also minimizing the number of unnecessary biopsies performed.

An overview of recent developments of electrochemical biosensors for multiplexed detection of PCa protein biomarkers is presented on Table 3.

### 3.3. Potential Companion Diagnostic Devices Using Integrated Biosensor Systems

Overall, the research prototypes discussed in this review have the potential to be just as accurate as traditional protein detection methods, and they can be implemented in conjunction with the ASSURED (Affordable, Sensitive, Specific, User-friendly, Rapid and Robust, Equipment-free, and Delivered to those who need it) strategy of companion diagnostics devices [132,133]. Thus, considerable attention has been dedicated to the creation of cost-effective integrated biosensor systems, such as the application of microfluidic systems, while also considering multiplexed detection techniques, as stated above. Microfluidic system integration is a crucial technique to explore for clinical usage by end users, as it can enable high-throughput detection while requiring smaller sample amounts, and possible automated control [89,134]. In addition, microfluidic systems can incorporate important steps performed in a clinical laboratory setting, such as sample preparation, molecular recognition, and signal amplification procedures (so-called lab-on-chip devices), negating the need for expensive specialist equipment or skilled professionals [135].

Key fabrication technologies used to detect PCa protein biomarkers included paper-based techniques, polymer (plastic-based) microfabrication, and the usage of microarrays [118]. When incorporating multiplexing approaches to either optical or electrochemical detection methods, each fabrication technology has some limitations, which can have an impact on translating prototypes into clinical utility that is appropriate for end users [89,136]. Because electrical instrumentation is difficult to incorporate, paper-based techniques such as LFIAs are more compatible with optical measurements. Although polymer (plastic-based) microfabrication designs are more efficient for electrochemical detection approaches, such platforms are not mass-produced in terms of electronics integration. As a result, the time and expense required to complete such processes significantly escalates [136]. Furthermore, increasing the number of spatially separated detection sites or regions for PCa protein biomarkers necessitates the inclusion of additional components such as more controls, valves, and detection/capture chambers, resulting in a complicated fabrication process. On the other hand, simple designs without controlled sites or regions could lead to cross-reactivity of sample solutions introduced to the biosensor system, resulting in unreliable detection results. Meanwhile, when using sandwich immunocomplexes as the surface chemistry, the inclusion of additional labels will necessitate additional washing steps in between incubation steps and/or detection measurements. Label-free surface chemistries, on the other hand, may impair detection sensitivity for certain PCa protein biomarkers with low serum concentrations. As a result, a compromise must be established between the integrated biosensor system’s complexity and the multiplexed detection approach [20,89].

Due to being mass-produced internationally at a cheap cost and being compact/portable, printed circuit boards (PCBs) could alleviate some of the issues faced with plastic-based biosensors for electrochemical biosensor systems [136,137]. Furthermore, PCBs have long been integrated with both electronic and microfluidic devices, requiring low sample volumes and coinciding with the ASSURED approach to xPOCT (lab-on-PCB approach) [136]. Therefore, multiplexing techniques combined with PCBs can potentially help secure consistent clinical outcomes for PCa patients that are highly sensitive and specific.

## 4. Conclusions and Future Perspectives

We summarized some of the emerging PCa protein biomarkers reported to date that may be relevant for PCa diagnosis, prognosis, and treatment monitoring, in conjunction with the widely known biomarker PSA. Although several markers have been recognized, these are still yet to be validated as PCa biomarkers [138]. In addition, this review looked at recent developments in miniaturized biosensor systems using optical and electrochemical detection techniques, to detect multiple PCa protein biomarkers simultaneously. Such miniaturized biosensor surfaces have been modified using a variety of labels or nanomaterials, such as enzymes, metallic nanoparticles, or graphene sheets, in order to achieve systems with multiplexing capacities. In addition, some of the biosensor platforms mentioned have been integrated with a microfluidic system, allowing the implementation of complex functions as seen in clinical laboratories (lab-on-a-chip approach). Thus, effectively achieving sensitive results while using smaller reagent and sample volumes in a reliable and accurate manner that is comparable to conventional clinical laboratory performance [139]. Additionally, the manufacture of 3D-printing microfluidic systems provides cost-effective and simple fabrication processes compared to conventional methods [52,99,108,139]. Moreover, analyzed results were well correlated with conventional methods, such as ELISA single-analyte kits. However, more work is needed to translate biosensor detection platforms into commercially available xPOCT devices in order to deliver highly cost-effective and sensitive results that leads to better management of PCa treatment in near-patient settings in a timely manner [20]. This would allow the use of multiplexed companion diagnostic devices as alternatives to conventional PCa diagnostic techniques, such as DRE, transrectal ultrasonography (TRUS), positron emission tomography (PET) and/or magnetic resonance imaging (MRI) [24].

Overall, recent developments showed that modifications to the surface of the biosensor can provide efficient and sensitive multiplexed protein detection. However, further research is needed to achieve simpler modification strategies that can still produce ultra-sensitive and clinically relevant detection of serum PCa-associated protein biomarkers. Increased availability of commercial multiplexed companion diagnostic devices could provide portable xPOCT devices in near-patient settings. For instance, bench-top analyzers or handheld devices that do not require highly trained professionals could be used within a primary clinical setting, e.g., a hospital or a GP clinic. If the established handheld devices are patient-friendly, it could be beneficial for both primary healthcare professionals, but also for patients to use at home [140]. Thus, developing xPOCT devices that achieve sensitive and specific results using a minute serum sample, and are comparable to the results evaluated in clinical laboratories using conventional single-analyte protein detection kits such as ELISA [20,141]. Moreover, efforts have been made to integrate wireless networks with xPOCT devices in order to effectively communicate and transfer real-time and highly sensitive results, aiding to prevent unnecessary misdiagnosis and unnecessary treatment [20,90,142]. Current research developments are focused on the detection of multiple proteins biomarkers in relation to early PCa screening and diagnosis. However, there is a greater need in the future for multiplexed PCa companion diagnostic devices to aid clinicians who need to make a conclusive decision on the ideal treatment pathway to be considered. Therefore, the provision of highly personalized approaches to PCa treatment management, particularly during key diagnostic, active surveillance or monitoring milestones while PCa patients are undergoing treatment, is in great need [21,140].

Many different methods have been identified in this review that could lead to multiplexed systems, some with extremely low limits of detection and/or high selectivity. However, each system would have advantages and drawbacks, and which would make it to market depends mostly on the clinical application: test by healthcare professional versus test at home, clinical ranges required, diagnostic or monitoring, number of proteins required to be measured, sample volumes available, cost, etc. It should also be noted that although we focused on protein biomarkers, a range of other biomarkers are of interest for PCa diagnosis and, in particular PCa prognosis such as microRNAs, circulating tumor DNA (ctDNA), circulating tumor cells (CTCs), etc. Several of the systems described could integrate true multiplexing capabilities by measuring different types of biomarkers.

## Figures and Tables

**Table 1:** Selected candidate serum protein biomarkers with the potential of being detected within the diagnostic, prognostic and/or predictive stages of PCa.

Protein	Type	Biological Characteristics	Clinical Relevance	Normal Serum Level	PCa Cut-Off Level	PCa Purpose	Sample	Ref
Alpha- Methylacyl- CoA Racemase (AMACR)	Peroxisomal and mitochondrial racemase	Involved in the body’s fatty acid synthesis and metabolism	Overexpression in prostate cancer (PCa) tissues Potential for distinguishing benign prostatic hyperplasia (BPH) patients from PCa patients	-	10.6 ng/mL	Diagnostic/Prognostic	Serum, Tissue and Urine	[3,43,46,47,48,49,50,51]
Cluster of Differentiation (CD) -14	Glycoprotein	Inflammation marker Presented either as a membrane bound CD-14 or in its soluble form	Increased expression leads to elevated concentration of monocyte in BPH tissues compared to PCa tissues Linked to advanced PCa	1.9 µg/mL	-	Diagnostic	Serum and Tissue	[52,53,54,55]
Chromogranin- A (CgA)	Pro-hormone peptide	A part of the granin family of proteins Released by neuroendocrine (NE) cells in the prostate gland and used as a cell marker for endocrine and NE cells	High CgA serum levels seen in patients with PCa-resistant hormone therapy compared to patients with BPH and indolent PCa. PCa patients with higher CgA levels have lower prognosis and survival compared to those with lower CgA levels	-	-	Diagnostic/Prognostic	Serum	[39,43,45,46,47,56]
Early Prostate Cell Antigen (EPCA) -1	Nuclear matrix protein	Uncertain contribution to nuclear morphology	Linked to early carcinogenesis and to predict repeated biopsy	-	-	Diagnostic	Serum and Tissue	[2,3,39,43,46,48]
EPCA -2	Nuclear matrix protein	Uncertain contribution to nuclear morphology	Connected to early carcinogenesis Can differentiate between indolent PCa and aggressive PCa	-	-	Diagnostic/Prognostic	Serum	[2,3,39,43,46,48]
Golgi Membrane protein (GOLM)-1	Type II Golgi membrane protein	Aids for the transport of proteins through the Golgi Apparatus. Also known as Golgi phosphoprotein 2 (GOLPH2) or (GP73)	Up-regulation in patients with PCa PCa gene fusion protein Suggested to be an aggressive PCa predictor	54 ng/mL	-	Diagnostic/Prognostic	Serum, Tissue, and Urine	[48,52,57,58,59]
Human Kallikrein 2 (hK2)	Serine protease enzyme	Secreted by epithelial cells in the prostate gland 80% homologous to PSA but different in enzymatic activity Splits pro-PSA, producing PSA Also known as Kallikrein-related peptide 2 (KLK2)	Highly expressed in prostate tissue, especially as PCa progresses to advanced stages Studies have shown strong correlation with PCa-specific survival, but large cohort validation studies required	-	-	Diagnostic/Prognostic	Serum and Tissue	[31,35,39,43,45,46,47,49,50,60]
Insulin-like Growth Factor-1 (IGF-1)	Growth hormone-dependent polypeptides	Produced alongside IGFBP-3 Involved in multiple cellular growth-related responses, including synthesis of DNA, RNA, and cellular proteins	Elevated serum levels Associated with cancer development during subclinical disease stages Not a useful biomarker for early diagnosis or screening of PCa	160 ng/mL	-	Diagnostic	Serum	[39,43,45,47,49,52,55,61,62,63,64]
IGF-Binding Protein-3 (IGFBP-3)	Binding protein	Produced alongside IGF-1 Binds to approximately 75 to 90% of circulating IGF-1, in conjunction with the acid-labile subunit	Under-expressed in PCa patients Suggested to limit the availability of IGF-1 and regulate apoptosis Suggested to be a strong predictor of significant PCa compared to indolent PCa	3.7 µg/mL	-	Diagnostic/Prognostic	Serum	[39,45,52,55,62,63,64]
Interleukin-6 (IL-6) and Receptor (IL-6R)	Cytokine	Has variable effects on immune and hematopoietic mechanisms Produced at acute and chronic inflammation sites	Elevated levels in PCa cells Associated with metastatic and androgen independent PCa Predictors of disease extent in the progression and survival of PCa patients Soluble IL-6R has a stronger correlation to disease progression than IL-6	0.006–0.02 ng/mL	0.02–1 ng/mL	Prognostic/Predictive	Serum	[2,31,43,47,48,65,66,67,68,69]
Platelet Factor-4 (PF-4)	Chemokine	Belongs to the chemokine (CXC) family, ligand 4 (CXCL4) Released during stimulation of the platelet	Lower serum levels in metastatic PCa patients, compared with healthy or indolent/clinically insignificant PCa patients	5–10 μg/mL in serum (2–10 ng/mL in plasma)	10–500 ng/mL	Diagnostic	Serum	[52,68,70,71,72,73,74]
Prostatic Acid Phosphatase (PAP)	Glycoprotein enzyme	Also known as Acpp or prostatic specific acid phosphatase (PSAP) More research is needed to fully understand the biological role	Elevated in patients with PCa metastasizing to the bone Potential biomarker for PCa progressions and response of advanced PCa to androgen deprivation therapy	-	>2.0 ng/mL	Diagnostic/Prognostic	Serum and Urine	[32,45,48,75,76,77]
Prostate-Specific Antigen (PSA)	Serine protease enzyme	Also known as Kallikrein-related peptidase 3 (KLK3) or human kallikrein 3 (hK3) Specifically produced by epithelial cells in the prostate gland Has the biological role of seminal fluid liquefaction Androgen-regulated by androgen response elements	Increased expression within the prostate gland Widely known PCa biomarker; however lacking in sensitivity and specificity	1–4 ng/mL	4.0 ng/mL	Diagnostic/Prognostic/Predictive	Serum and Urine	[35,39,45,47,48,52,68,69,75,78]
Prostate-Specific Membrane Antigen (PSMA)	Type II integral membrane glycoprotein with cell surface carboxypeptidase function	Expressed in the epithelial cells of the prostate Also known as Folate hydrolase 1, which has the function of folate hydrolase Involved in cell stress reaction, signal transduction, cell migration, and nutrient uptake	Highly expressed in PCa cells compared to BPH and normal cells Suggested role in PCa progression	200–300 ng/mL	300–650 ng/mL [68,69] or 349.4–946.6 ng/mL [79]	Diagnostic/Prognostic	Serum and Urine	[2,43,46,47,48,49,52,60,68,69,79]
Prostate Stem Cell Antigen (PSCA)	Membrane glycoprotein	Specifically produced in the prostate gland Involved in the regulation of cell proliferation	Possible therapy target Increased expression associated with higher Gleason score, higher stage and in the presence of aggressive PCa	-	-	Prognostic	Serum and Tissue	[2,46,48,49]
Testosterone	Steroid hormone	Involved in the development and preservation of prostate gland and seminal vesicles Significant role in sexual development and anabolism Acts on endocrine signal transduction	Androgen receptor ligand associated with. The spread of PCa Higher levels in aggressive than indolent cancer patients More research needed to fully understand its clinical relevance	-	-	Prognostic/Predictive	Serum	[43,45,46,78]
Transforming Growth Factor- ß1 (TGF-ß1)	Cytokine	Growth factor Involved in cell proliferation, immune response, differentiation, and angiogenesis	Suggested promotion of PCa cell progression Associated with higher tumor grade, tumor invasion and metastasis in PCa patients	-	-	Prognostic	Serum	[2,31,43,47,49,50]
Urokinase Plasminogen Activator (uPA) and Receptor (uPAR)	Serine protease and transmembrane receptors	Plasminogen activator, converting plasminogen into plasmin Plasmin activates protases associated with the degradation of extracellular matrix	Highly expressed in PCa and BPH patients compared to healthy patients Correlated with aggressive PCa recurrences Suggested as a predictor of biochemical progression following surgery	-	-	Diagnostic/Prognostic	Serum and Tissue	[2,31,39,43,47,48,50,80,81]
Vascular Endothelial Growth Factor (VEGF)	Dimeric, heparin-binding protein	Vital endothelial cell growth factor in controlling the angiogenesis of the tumor and increase vascular permeability	Elevated concentration in PCa patients Suggested PCa angiogenesis	657 (±43) pg/mL for VEGF-D ligand	-	Prognostic	Serum	[43,52,55,66,82,83]

**Table 2:** Overview of recent developments of optical biosensors for multiplexed detection of PCa protein biomarkers with indication of tests done with PCa patient samples.

Technique	Biomarkers	Sensor Surface Modification	Detection Label	Linear Detection Range	Limit of Detection	Ref
Fluorescence (PCa patient samples)	fPSA	Ab_1_ (monoclonal tPSA capture antibody)	For fPSA: Ab2/MQB625	–	fPSA: 0.009 ng/mL	[95]
	cPSA		For cPSA: Ab2/MQB525		cPSA: 0.087 ng/mL	
CL (PCa patient samples)	PSA	Ab_1_	Ab_2_/poly HRP	PSA: 0.5 pg/mL–5 ng/mL *	0.5 pg mL−1 for both PSA and PF-4	[99]
	PF-4			PF-4: 0.5 pg/mL–10 ng/mL *		
CL	fPSA fPSA glycans	GOPTS/Apt	For fPSA: Ab_2_ (Anti-fPSA)/HRPFor fPSA glycans: biotinylated SNA/SA-HRP	fPSA: 0.01 to 50 ng/mL	fPSA: 0.5 ng/mL	[100]
				fPSA glycans: 3 to 50 ng/mL	fPSA glycans: 3 ng/mL	
CL (PCa patient samples)	fPSA tPSA	Ab_1_ (monoclonal tPSA capture antibody)	For tPSA & fPSA: Ab2/HRP	–	fPSA: 0.03 ng/mL	[102]
			For fPSA: Ab3/ALP		tPSA: 0.05 ng/mL	
ECL (PCa patient samples)	PSA	SWCNT/Ab_1_	Ab2/RuBPY-SiNP	PSA: 1 pg/mL–10 ng/mL *	PSA: 1 pg/mL	[105]
	IL-6		(PSA and IL-6 conjugated to the same RuBPY-SiNPs)	IL-6: 0.1 pg/mL–2 ng/mL *	IL-6: 0.25 pg/mL	
ECL (PCa patient samples)	PSA	SWCNT/Ab_1_	Ab2/RuBPY-SiNP	PSA: 100 fg/mL–40 pg/mL (100 fg/mL–10 ng/mL *)	PSA: 100 fg/mL	[106]
	IL-6		(PSA and IL-6 conjugated to the same RuBPY-SiNPs)	IL-6: 0.5 fg/mL–10 fg/mL (0.5 fg/mL–1 ng/mL *)	IL-6: 10 fg/mL	
ECL (PCa patient samples)	PSA	SWCNT/Ab_1_	For Label 1 (PSA & IL-6) and Label 2 (PSMA & PF-4): Ab_2_/RuBPY-SiNP	PSA: 100 fg/mL–1 ng/mL *	PSA: 50 fg/mL	[107]
	PSMA			PSMA: 100 fg/mL–10 ng/mL *	PSMA: 100 fg/mL	
	PF-4			PF-4: 100 fg/mL–5 ng/mL *	PF-4: 10 fg/mL	
	IL-6			IL-6: 100 fg/mL–5 ng/mL *	IL-6: 10 fg/mL	
ECL (PCa patient samples)	PSA	SWCNT/Ab_1_	Ab_2_/RuBPY-SiNP	For all proteins: 500 fg/mL–10 ng/mL *	PSA: 300 fg/mL	[108]
	PSMA				PSMA: 535 fg/mL	
	PF-4				PF-4: 420 fg/mL	
ECL (PCa patient samples)	PSA	SWCNT/Ab_1_	For Label 1 (PSA & PSMA), label 2 (VEGF-D & PF-4), label 3 (CD-14 & IGF-1), and label 4 (GOLM-1 & IGFBP-3): Ab_2_/RuBPY-SiNP	For all proteins: 0.5 pg/mL–10 ng/mL	For all proteins: 110–500 fg/mL	[52]
	PSMA					
	VEGF-D					
	PF-4					
	CD-14					
	IGF-1					
	GOLM-1					
	IGFBP-3					
SERS (PCa patient samples)	PSA	SiC/Ag-AgNPs/Ab_1_	AgNPs/4-MBA/Ab_2_	PSA: 0.46 fg/mL–478.93 ng/mL	PSA: 0.46 fg/mL	[60]
	PSMA			PSMA: 1.05 fg/mL–113.4 ng/mL	PSMA: 1.05 fg/mL	
	hK2			hK2: 0.67 fg/mL–466.23 ng/mL	hK2: 0.67 fg/mL	
SERS (PCa patient samples)	PSA	Ab_1_	For PSA: AuNBA-Ag/Ab2	PSA: 1 pg/mL–10 µg/mL	PSA: 0.37 pg/mL	[115]
	CEA		For CEA: Au4-MB-Ag/Ab2	CEA: 10 pg/mL–1 µg/mL	CEA: 0.43 pg/mL	
	AFP		For AFP: Au4-NBT-Ag/Ab2	AFP: 10 pg/mL–1 µg/mL	AFP: 0.26 pg/mL	
SERS (PCa patient samples)	PSA	Ab_1_	AuNRs-DTNB/Ab_2_/BSA	-	For all proteins: 10 pg/mL	[114]
	CEA					
	AFP					

Abbreviations: 4-MB = 4-Mercaptobenzonitrile, 4-MBA = 4-mercaptobenzoic acid, 4-NBT = 4-nitrobenzenethiol, Ab_1_ = capture antibody, Ab_2_ = detection antibody, Ab_3_ = secondary detection antibody, AFP = α-1-fetoprotein, AgNP = silver nanoparticles, ALP = alkaline phosphatase, Apt = Deoxyribonucleic acid aptamer, BSA = Bovine Serum Albumin, CD-14 = Cluster of differentiation-14, CEA = Carcinoembryonic antigen, CL = Chemiluminescence, cPSA = complexed PSA, DTNB = 5,5′-dithiobis-(2-nitrobenzoic acid), ECL = Electrochemiluminescence, fPSA = free PSA, GOLM-1 = Golgi membrane protein-1, GOPTS = (3-Glycidyloxypropyl) trimethoxysilane, hK2 = Human kallikrein 2, HRP = horseradish peroxidase, IGF-1 = Insulin-like Growth Factor-1, IGFBP-3 = Insulin-like Growth Factor binding protein-3, IL-6 = Interleukin-6, MQB = magnetic-quantum dot nanobeads, NBA = Nile Blue A, PF-4 = Platelet Factor-4, PSMA = Prostate-specific membrane antigen, RuBPY-SiNP = Tris(bipyridine)ruthenium(II) chloride, SA = Streptavidin, SERS = Surface-enhanced Raman scattering, SiNP = silica nanoparticles, SNA = Sambucus nigra, SWNCT = single-wall carbon nanotube, tPSA = total PSA, VEGF-D = Vascular endothelial growth factor. * Dynamic detection ranges.

**Table 3:** Overview of recent developments of electrochemical biosensors for multiplexed detection of PCa protein biomarkers with indication of tests done with PCa patient samples.

Technique	Biomarkers	Sensor surface modification	Detection Label	Linear Detection Range	Limit of Detection	Ref
Amp (PCa patient samples)	PSA	For PSA & PSMA: SWCNF/Ab_1_ For PF-4 & IL-6: SWCNF/Ab_1_	For PSA & PSMA: Ab_2_/HRP For PF-4 & IL-6: Ab_2_/SA-HRP	PSA: 1–40 ng/mL	PSA: 1 ng/mL	[68]
	PSMA			PSMA: 10–250 ng/mL	PSMA: 10 ng/mL	
	PF-4			PF-4: 1–40 ng/mL	PF-4: 1 ng/mL	
	IL-6			IL-6: 50–500 pg/mL (biphasic with better sensitivity below 350 pg/mL)	IL-6: 0.03 ng/mL	
Amp(PCa patient samples)	PSA	GSH-AuNPs/Ab_1_	MP/Ab_2_/HRP	–	PSA: 0.23 pg/mL	[15]
	IL-6				IL-6: 0.30 pg/mL	
Amp(PCa patient samples)	PSA	ERGO/Ab_1_	Ab_2_/Fe_3_O_4_ NPs/GO	PSA: 61 fg/mL–3.9 pg/mL *	PSA: 15 fg/mL	[119]
	PSMA			PSMA: 9.8 fg/mL–10 pg/mL *	PSMA: 4.8 fg/mL	
Amp	PSA	PDDA/GSH-AuNPs/Ab_1_	MP/Ab_2_-HRP	PSA: 0.14–34.2 ng/mL	PSA: 140 pg/mL	[55]
	VEGF			VEGF-D: 0.09–23.8 ng/mL	VEGF-D: 90 pg/mL	
	ERG			ERG: 0.015–3.9 ng/mL	ERG: 15 pg/mL	
	IGF-1			IGF-1: 0.013–3.4 ng/mL	IGF-1: 13 pg/mL	
	IGFBP-3			CD-14: 0.13–32.5 ng/mL	CD-14: 130 pg/mL	
	CD-14			IGFBP-3: 0.15–38.7 ng/mL	IGFBP-3: 150 pg/mL	
	PEDF			PEDF-1: 0.09–11.2 ng/mL	PEDF-1: 90 pg/mL	
	GOLM-1			GOLM-1: 0.015–1.95 ng/mL	GOLM-1: 15 pg/mL	
DPV	PSA	SAM(MPA)/Ab_1_	MP/Ab_2_/HRP	PSA: 2 pg/mL–200 ng/mL *	0.05–2 pg/mL	[123]
	PSMA			PSMA: 0.15 pg/mL–15 ng/mL *		
	PF-4			PF-4: 0.1 pg/mL–10 pg/mL *		
	IL-6			IL-6: 0.05 pg/mL–5 ng/mL *		
DPV (PCa patient samples)	PSA	GO/Apt	For VEGF & PSA: Ab_2_/PPLA NPs, where Ab_2_ is a mixture of anti-VEGF and anti-PSA antibodies	–	PSA: 1 ng/mL	[124]
	VEGF				VEGF: 50 pg/mL	
SWV (PCa patient samples)	tPSA	GO/AuNPs/Ab_1_	GO/AuNPs/Ab_2_	–	tPSA: 0.2 ng/mL fPSA: 0.07 ng/mL	[125]
	fPSA					
CV	PSA	Ab_1_/MBs	HRP/Ab_2_/AuNRs	PSA: 0.1–10 ng/mL	PSA: 0.1 ng/mL	[69]
	PSMA			PSMA: 0.8–400 ng/mL	PSMA: 0.8 ng/mL	
	IL-6			IL-6: 5–1000 pg/mL	IL-6: 0.005 ng/mL	
EIS	fPSA	SAM (MUA-2-ME)/Ab_1_	–	–	1 ng/mL	[129]
	tPSA					
EIS	PSA	SAM (MUA-MCH)/Ab_1_	SNA	4 a.m. to 40 nM	PSA: 4 aM	[130]
	PSA glycans				PSA glycans: down to 4 a.m. (~0.13 fg/mL)	
EIS (PCa patient samples)	PSA	SAM(AUT)/MCH/AuNPs/SAM(Apt-MCH)		PSA: 0.64–62.5 ng/mL *	PSA: 0.64 ng/mL	[131]
	PSA glycans			PSA glycans: 0.26–62.5 ng/mL *	PSA glycans: 0.26 ng/mL	

Abbreviations: 2-ME = 2-mercaptoethanol, Ab_1_ = capture antibody, Ab_2_ = detection antibody, Amp = Amperometry, Apt = DNA aptamer, AuNPs = Gold nanoparticles, AuNRs = Gold nanorods, AUT = 11-amino-1-undecanothiol, CD-14 = Cluster of differentiation-14, CV = Cyclic Voltammetry, DPV = Differential Pulse Voltammetry, EIS = Electrical Impedance Spectroscopy, ERG = Erythroblast transformation specific related gene, ERGO = electrochemically reduced graphene oxide, Fe_3_O_4_ NPs = Iron oxide nanoparticles, fPSA = Free PSA, GO = Graphene oxide, GOLM-1 = Golgi membrane protein-1, GSH = Glutathione, HRP = horseradish peroxidase, IGF-1 = Insulin-like Growth Factor-1, IGFBP-3 = Insulin-like Growth Factor binding protein-3, IL-6 = Interleukin-6, MB = magnetic beads, MCH = 6-mercapto-1-hexanol, MP = magnetic nanoparticles, MPA = mercaptopropionic acid, MUA = 11-mercaptoundecanoic acid, PDDA = poly(diallyl dimethylammonium chloride), PEDF = Pigment epithelium-derived factor, PF-4 = Platelet factor-4, PPLA NPs = Poly-L-lactide nanoparticles, PSA = Prostate-Specific Antigen, PSMA = Prostate-specific membrane antigen, SA = streptavidin, SAM = self-assembly monolayer, SNA = Sambucus nigra, SWCNF = single-wall carbon nanotube forests, SWV = Square Wave Voltammetry, tPSA = Total PSA, VEGF = Vascular endothelial growth factor. * Dynamic detection ranges.

## Data Availability

Not applicable.
